# A Research Agenda for Malaria Eradication: Vaccines

**DOI:** 10.1371/journal.pmed.1000398

**Published:** 2011-01-25

**Authors:** The malERA Consultative Group on Vaccines

## Abstract

Vaccines could be a crucial component of efforts to eradicate malaria. Current attempts to develop malaria vaccines are primarily focused on *Plasmodium falciparum* and are directed towards reducing morbidity and mortality. Continued support for these efforts is essential, but if malaria vaccines are to be used as part of a repertoire of tools for elimination or eradication of malaria, they will need to have an impact on malaria transmission. We introduce the concept of “vaccines that interrupt malaria transmission” (VIMT), which includes not only “classical” transmission-blocking vaccines that target the sexual and mosquito stages but also pre-erythrocytic and asexual stage vaccines that have an effect on transmission. VIMT may also include vaccines that target the vector to disrupt parasite development in the mosquito. Importantly, if eradication is to be achieved, malaria vaccine development efforts will need to target other malaria parasite species, especially *Plasmodium vivax*, where novel therapeutic vaccines against hypnozoites or preventive vaccines with effect against multiple stages could have enormous impact. A target product profile (TPP) for VIMT is proposed and a research agenda to address current knowledge gaps and develop tools necessary for design and development of VIMT is presented.

## Abstract 2

The Malaria Eradication Research Agenda (malERA) Consultative Group on Vaccines present a research and development agenda to provide the tools to create vaccines that may be used during malaria eradication.

**Summary Points**

- Vaccines for malaria eradication need to have an impact on transmission rather than focusing on mortality and morbidity reduction alone
- Vaccines that interrupt malaria transmission (VIMT) may target many stages of the parasite’s life cycle, not just the sexual and mosquito stages as in classical blocking vaccines and multiple plasmodium species, in particular *Plasmodium vivax*
- Novel vaccine delivery approaches and adjuvants need to be developed
- Other priority areas for research and development include the development of tools to measure transmission rates and the development of robust assays of functional immune responses in individuals, which could inform vaccine development
- A better understanding of the dynamics between the multiplication of parasites, gametocytogenesis, and malaria transmission rates in populations is also needed

## Introduction

Vaccines are the most cost-effective tools for public health and have been instrumental in previous elimination campaigns against smallpox [1], polio [2], and measles [3],[4]. Vaccines have also been useful for sustained control of diseases such as neonatal tetanus [5], and vaccines such as *Haemophilus influenzae* type b conjugate vaccine have the potential to lead to elimination in some settings [6].

Here, we discuss the research and development agenda for the development of vaccines that can serve as key components of a future arsenal of tools to eradicate malaria. Current efforts to develop malaria vaccines are primarily directed towards reducing the morbidity and mortality that are associated with malaria and focus on *P. falciparum*. For example, the Malaria Vaccine Roadmap [7] has a strategic goal of developing a vaccine with 80% protective efficacy against *P. falciparum* by 2020. However, if malaria vaccines are to contribute to programs for malaria elimination, they will need to have an impact on malaria transmission. The scientific and ethical basis for the development of vaccines referred to as transmission-blocking vaccines (TBVs) that specifically target malaria sexual stage antigens with the goal of having an impact on transmission has been described previously [8],[9]. Here, we refocus attention on the development of vaccines that can be used in concert with other malaria control interventions to interrupt malaria transmission and eventually contribute to the eradication of this disease. We also recommend that vaccine development efforts need to pay attention to *Plasmodium* species other than *P. falciparum*, especially *Plasmodium vivax*, if malaria eradication is to be achieved.

## Rationale of the Proposed malERA Approach to Development of Malaria Vaccines

First, we introduce the broad concept of VIMT. VIMT may be composed of one or more of the following components: classical TBVs that target sexual and mosquito stage parasite antigens; highly effective pre-erythrocytic vaccines that reduce asexual and sexual stage parasite prevalence rates; highly effective asexual erythrocytic stage vaccines that inhibit multiplication of asexual stage parasites efficiently to reduce blood-stage parasite densities and have an impact on malaria transmission; and vaccines that target vector antigens to disrupt parasite development in the vector. It seems obvious that a highly effective pre-erythrocytic vaccine that prevents erythrocytic stage infection will reduce transmission, but the effect of partially effective pre-erythrocytic or asexual blood-stage vaccines on individual infectivity needs investigation. A successful VIMT must primarily reduce malaria transmission. However, VIMTs that include pre-erythrocytic and/or asexual blood-stage vaccine components may also provide individuals with protection against malaria. Such VIMT would also protect the population against epidemic spread following reintroduction of malaria after elimination, an important characteristic given that the gains accrued through many years of elimination can be rapidly reversed if malaria is reintroduced to a population with no antimalarial immunity [10].

Second, the observed impact of concerted nonvaccine malaria control efforts on transmission dynamics in several malaria-endemic regions has shown that high-intensity transmission settings (entomological inoculation rate, EIR >50) can be converted to low-to-moderate intensity transmission settings (EIR <10) [11],[12]. Implementation of VIMT together with such control efforts may successfully drive down transmission rates to reduce the effective reproduction rate (*R*_effective_) to below 1.0.

Third, the consultative group introduces the concept of a detailed TPP for this class of vaccines and urges that novel clinical development methods and approaches be considered to shorten the time to VIMT registration and implementation.

Fourth, the consultative group lays out a detailed research agenda that must be developed, funded, and implemented in parallel with VIMT development efforts. This agenda includes development of critical tools that will be required to register and implement such a vaccine. In particular, we identify the need to develop robust assays to measure biologically relevant transmission-blocking activities at the individual level that are validated as surrogates of reductions in transmission rates at the population level. If this goal is achieved, such assays could become the key tool for measurement of primary vaccine efficacy endpoints in conditional registration trials, thereby simplifying the clinical development program.

Finally, the consultative group considers that interested industrial partners should be identified early on in development, because expertise in applied immunology, vaccinology, product development, manufacturing, and regulatory activities is concentrated within industry and will play an essential role in the successful development of VIMT. In addition, it will be important to engage with regulatory agencies to define efficient yet sound regulatory strategies to develop and register new tools that can meet the needs of global malaria elimination and eradication efforts.

## TPP for VIMT

A TPP is an industry-standard tool that gives clear guidance on the critical characteristics of a candidate product under development. TPPs are developed early in the development process and ensure that research and development efforts are focused on those activities that are necessary to develop a product that will meet the needs of end users. Table 1 presents a TPP for VIMT. For each characteristic in this TPP, we propose a “desired target” (aspirational) and a “minimally acceptable target” (must achieve). A vaccine candidate that does not meet or exceed most, if not all, of the minimally acceptable targets is likely to have a significantly reduced likelihood of successful introduction and uptake.

**Table 1:** TPP VIMT.

Item	Desired Target	Minimally Acceptable Target
**Indication**	The candidate vaccine is indicated for active immunization of individuals for protection against *P. falciparum* and *P. vivax* malaria and to achieve reduction of transmission rates of all strains of *P. falciparum* and *P. vivax* so that *R*_effective_ <1^a^.	The candidate vaccine is indicated for active immunization of individuals to achieve reduction of transmission rates of all strains of *P. falciparum* so that *R*_effective_ <1^a^ in conjunction with other control measures.
**Target populations**	The vaccine can be administered to all age groups and populations, including pregnant women, persons with immunodeficiencies, malnourished individuals, or otherwise high risk populations.	The vaccine can be administered to otherwise healthy persons who may transmit malaria, including infants, children, adolescents, and adults in malaria-endemic regions.
**Route of administration**	The vaccine is administered orally or by intramuscular or subcutaneous injection or by other innovative device.	The vaccine is administered by intramuscular, intradermal subcutaneous injection, or an innovative device.
**Product presentation**	The vaccine is available in a single dose auto-disposable compact prefilled device. Low multidose presentations (ten doses/vial) are also needed.	The vaccine is provided as a lyophilized or liquid product in single dose vials or an auto-disposable compact prefilled device; or low-dosage (two doses) vials that may be accompanied by a separate paired vial containing adjuvant/diluents. A suitable preservative may be required for multidose vials. Reconstitution may be required prior to administration.
**Dosage schedule**	A single dose vaccine that can be administered by either mass administration or clinic-based programs. Booster dose may be required after 2 years.	A maximum of two to three doses of vaccine that can be administered according to a schedule feasible for both mass administration and clinical-based programs. A booster dose may be necessary 4–6 months after the second dose and after 2 years.
**Warnings and precautions/pregnancy and lactation**	The vaccine has a safety and reactogenicity profile comparable to hepatitis B vaccine. The vaccine can be safely administered to pregnant women. There should be no increased risk of autoimmune or other chronic diseases related to vaccination.	In young children, the vaccine has a similar safety and reactogenicity profile to currently administered combination vaccines such as DTPwHepBHib administered through EPI. In adults, the vaccine has a similar safety and reactogenicity profile as hepatitis B vaccine or tetanus toxoid. The vaccine can be safely administered to pregnant women. There should be no increased risk of autoimmune or other chronic diseases related to vaccination.
**Expected efficacy**	Reduces *R*_effective_ below 1.0 in a malaria-endemic population and provides protection against *P. falciparum* and *P. vivax* for at least 2 years.	When used in a malaria-endemic population that employs ITNs, IRS, or other malaria control tools, further reduces *R*_effective_ to below 1.0 for at least 1 year.
**Coadministration**	The vaccine can be coadministered with any licensed vaccine without a clinically significant interaction in relation to safety or immunogenicity. For use in infants with other EPI vaccines, specific coadministration studies must be completed to demonstrate the noninferiority of responses to EPI vaccines given in coadministration.	The vaccine will be given as a stand-alone product not coadministered with other vaccines.
**Shelf life**	The product must have a minimum shelf life of 36 months and a Vaccine Vial Monitor should be attached (see [54]).	The product must have a shelf life of at least 24 months and a Vaccine Vial Monitor should be attached (see [54]).
**Storage**	The product must be stable at ambient temperature and withstand freeze thawing.	At a minimum, vaccines should be stable at refrigerated storage temperatures (2–8°C). New vaccines should be formulated to maximize heat stability to improve effectiveness in light of the challenges faced in distributing vaccines in developing countries. Vaccine vial monitors should be included on all vaccines in accordance with the WHO and UNICEF joint policy statement and the WHO prequalification standards for vaccines. In case of live, attenuated sporozoite vaccines, vaccine should be stable at −70°C.
	Vaccine vial monitors should be included on all vaccines in accordance with the WHO and UNICEF joint policy statement and the WHO prequalification standards for vaccines.	
**Product registration and WHO prequalification**	Product must be WHO prequalified (see [54]) and registered with EMEA and FDA.	Conditional registration or recommendation by WHO or competent NRA followed by a large impact study in phase IV.
		Product must be WHO prequalified (see [55]).

a *R*_effective_, number of individuals who can be infected from a single untreated malaria case in an endemic area.

EMEA, European Medicines Agency; EPI Expanded Programme on Immunization; FDA, US Food and Drug Administration; NRA, National Regulatory Agency; IRS, indoor residual insecticide spraying; ITN, insecticide-treated net.

*P. falciparum* and *P. vivax* are the two most common *Plasmodium* species that cause human malaria. *P. falciparum* is responsible for most malaria-related deaths. As a result, previous efforts to develop vaccines for malaria have focused on *P. falciparum*, which causes ∼500 million cases of malaria annually and is critically important for Africa. However, *P. vivax* causes significant morbidity in other regions of the world including South and Southeast Asia and Latin America with around 75–90 million cases of *P. vivax* malaria reported annually [13]. Recent clinical epidemiology studies have confirmed that *P. vivax* can cause severe disease and may also contribute to malaria-associated mortality [14]–[17]. Efforts to eliminate malaria outside Africa must therefore address both parasite species. Ideally, VIMT should reduce transmission rates so that *R*_effective_ for both *P. falciparum* and *P. vivax* is driven to less than 1 and should provide protection against clinical malaria caused by both parasite species. At a minimum (and possibly more realistically), VIMT should achieve reduction of transmission rates (*R*_effective_ <1) of at least all *P. falciparum* strains leading to elimination of *P. falciparum* when used in conjunction with other control measures in elimination/eradication campaigns.

As better control is achieved, exposure to malaria parasites will decrease and “naturally acquired” immunity may play a diminished role. The mechanisms of clinical immunity observed in populations under high exposure may have little relevance as, increasingly, most infections will occur in people with little previous exposure. Therefore, our TPP specifies that a vaccine intended to interrupt transmission should not presume an age-specific risk or preexisting state of immunity against malaria disease or transmission. It is likely that VIMT may need to be implemented in the entire population.

Other ideal as well as minimally acceptable parameters for VIMT include product presentation, dosage, storage, and coadministration with other immunizations. These parameters are detailed in Table 1.

## Research in Support of Development of VIMT

Much of the ongoing work on malaria vaccine development has focused on the development of interventions that address disease manifestations and the work has been primarily focused on *P. falciparum*. To support the development of vaccines and other tools necessary for malaria eradication new dimensions need to be added to the fundamental research portfolio (see [18] also). For example, *P. vivax* needs to be added, and efforts need to be refocused on the development of vaccines that target sexual and mosquito stages of malaria parasites, which should interrupt transmission. The expanded portfolio also needs to include more research on vaccine delivery systems and adjuvants, the transmission dynamics and population biology of malaria parasites, and measurements of transmission rates.

### Human Malaria Parasites beyond *P. falciparum*

VIMT that target *P. falciparum* alone are likely to be deployed only in regions where *P. falciparum* is the species predominantly responsible for malaria. Regions where *P. vivax* is responsible for a significant proportion of the malaria burden will require VIMT that target both species.

Control efforts in regions where *P. falciparum* and *P. vivax* both occur indicate that it is more difficult to reduce transmission of *P. vivax* than of *P. falciparum* This increased difficulty is attributed in part to the development of gametocytes earlier during blood-stage infections with *P. vivax* than is the case for *P. falciparum*, which allows transmission before clinical symptoms are apparent. Other factors contributing to the difficulty of reducing *P. vivax* transmission include: the development of hypnozoites that remain latent in hepatocytes and lead to blood-stage infections months or even years later; transmission by outdoor biting mosquitoes; and the ability of *P. vivax* to complete its life cycle in a wider range of climatic and ecological conditions than *P. falciparum*. Because of these unique features of *P. vivax*, traditional malaria control efforts such as vector control, bednets, and early detection and treatment often fail to control *P. vivax* transmission. Vaccines that elicit long-lasting immune responses that prevent infection or inhibit gametocyte development or transmission of sexual stages are likely to be more effective tools for control of *P. vivax*. Given that latent hypnozoites can lead to blood-stage infections years after an infective bite, it may be necessary to continue deployment of VIMT that target *P. vivax* after elimination is achieved. An alternative would be to develop vaccine components that can target and eliminate hypnozoites. Design of such vaccines will require better understanding of the unique aspects of the biology of *P. vivax* hypnozoites at the molecular level.

Other *Plasmodium* species such as *Plasmodium ovale* and *Plasmodium malariae* account for less than 5% of malaria cases worldwide. Natural infection of humans by *Plasmodium knowlesi* has recently been reported [19],[20]. Thus, we need to be prepared for the emergence of new *Plasmodium* species that can cause human malaria. It remains to be seen whether these parasite species will survive once efforts to eliminate *P. falciparum* and *P. vivax* are successful. For now, then, efforts should be focused on developing VIMT for *P. falciparum* and *P. vivax* malaria, but it will be important to monitor the epidemiology of *P. ovale*, *P. malariae*, and *P. knowlesi* as elimination of *P. falciparum* and *P. vivax* progresses. Decisions to support development of vaccines that block transmission of these parasite species may need to be made in the future.

### Discovery Research

Malaria parasites have a complex life cycle during which they infect humans and are transmitted by Anopheline mosquitoes. The successful completion of the parasite life cycle requires specific molecular interactions between the parasite and various host and vector tissues. A clear understanding of the molecular interactions that mediate invasion of hepatocytes by Plasmodium sporozoites, invasion of erythrocytes by *Plasmodium* merozoites, and traversal of mosquito midgut epithelium by *Plasmodium* ookinetes may allow the development of strategies to target these key interactions and disrupt the parasite life cycle thereby reducing malaria transmission rates. It may be necessary to combine components that target different stages of malaria parasites to achieve synergistic effects that provide protection and reduce malaria transmission rates. For example, partially effective pre-erythrocytic and blood-stage components may not have any effect on transmission but the addition of such partially effective components to classical TBVs might allow the development of a multicomponent VIMT that can reduce malaria transmission as well as provide protection against malaria.

### Targeting the Sexual and Mosquito Stages

Gametocytes are the source of the epidemiologically important transmission of all malaria parasites. In *P. falciparum*, recent work has demonstrated that the developmental switch from asexual replication to sexual stage development occurs at the ring stage and that all schizonts from that ring parasite are committed to form gametocytes upon invasion of new red blood cells [21]. *P. falciparum* then undergoes sequential development through five distinct morphological stages to form mature male and female gametocytes. Within the mosquito midgut, mature male and female gametes are released and fertilization occurs to form a zygote. The resultant motile ookinete passes through the midgut wall, undergoes reduction division, and forms an oocyst. Each step in this developmental pathway involves unique processes, including the transcription of specific genes, the expression of specific proteins, the upregulation of specific biochemical pathways, and the formation of new morphological structures. Understanding the regulation of this developmental process could be the key to developing new interventions that target sexual and mosquito stages to interrupt transmission. For example, direct targeting of the developing gametocyte has the potential advantage of targeting a small subset of infected red blood cells that express proteins or pathways specific to parasite sexual development. A drug or a vaccine that could inhibit the initial switch to sexual development, coupled with a vaccine that targets gamete antigens might provide a powerful combinatorial approach to reduce transmission (also see [22]).

There is a large body of work on the key antigens on the surface of gametes of both *P. falciparum* and *P. vivax* [9]. Several of these antigens have been tested in animal models as transmission-blocking vaccines, at least two which have been tested in humans [23],[24]. A phase I trial of the *P. vivax* ookinete surface antigen Pvs25 formulated with Alhydrogel demonstrated acceptable safety and reactogenicity with induction of anti-Pvs25 immunoglobulin G (IgG) with functional transmission-blocking activity in a membrane-feeding assay. However, these data suggest that a more immunogenic formulation would be desirable to achieve higher transmission-blocking activity [23]. More recently, a trial of ISA51 formulations of Pvs25 and Pfs25 was terminated because of unacceptable reactogenicity [24]. The expression of correctly folded Pfs48/45 gametocyte surface antigen has recently resulted in a demonstration of transmission-reducing activity in sera from immunized animals [25],[26].

### Targeting Pre-erythrocytic and Asexual Stages

Highly effective pre-erythrocytic stage vaccines can, in principle, reduce the prevalence of blood-stage parasites, including both the asexual stages and the gametocytes. Such vaccines can provide protection against malaria and reduce malaria transmission. Immunization with irradiated sporozoites has elicited complete protection against sporozoite challenge in experimental animal models and in humans. Thus, in principle, it should be possible to target pre-erythrocyte stage antigens to elicit complete protection against parasite infection. Protective immune mechanisms elicited by irradiated sporozoites are not well understood but are thought to include antibody responses against sporozoite antigens that prevent hepatocyte infection, and cellular responses that clear infected hepatocytes. Better understanding of the correlates of immunity elicited by immunization with irradiated sporozoites could guide the development of highly effective pre-erythrocytic subunit vaccines that both provide protection and reduce parasite transmission. A recombinant vaccine based on the circumsporozoite protein, RTS,S has been shown to elicit partial protection against *P. falciparum* infection [27],[28]. It seems unlikely, however, that RTS,S will have significant impact on gametocyte prevalence or affect malaria transmission.

Other vaccines based on irradiated sporozoites or genetically modified attenuated sporozoites have provided protection in challenge models [29],[30]. Such whole organism attenuated vaccines may provide effective protection against malaria and significantly reduce parasite transmission. However, considerable technological challenges in terms of manufacturing, formulation, and delivery of such attenuated sporozoite vaccines need to be overcome.

During *P. vivax* infections, some infected hepatocytes differentiate into latent hypnozoite stages that can yield merozoites after a long latency period. The biology of hypnozoites is very poorly understood but the development of drugs or vaccines that can clear hypnozoites is critical for success of efforts to eradicate *P. vivax* [22]. The development of methods for *in vitro* culture of hypnozoites could greatly help improve our understanding of this latent stage. *In vitro* culture of hypnozoites would allow the application of whole genome approaches such as transcriptomics and proteomics to the identification of parasite proteins expressed in hypnozoites. It may be possible to elicit cellular immune responses against such hypnozoite specific proteins to clear these latent stages. Vaccines against pre-erythrocytic stages of *P. vivax* that are effective against both developing and resident hypnozoites would be of inestimable benefit in efforts to eliminate *P. vivax.*

Vaccines based on asexual blood-stage antigens may be effective at reducing parasite densities and provide protection against clinical disease but it is not clear whether such vaccines can reduce malaria transmission rates effectively. Basic research is needed to understand the dynamics of the relationship between asexual stage parasite growth, sexual stage parasite densities in blood, and individual infectivity or transmission efficiency. Recombinant vaccines based on asexual blood-stage antigens tested in human clinical trials have not yielded high rates of growth inhibition thus far and are unlikely to have significant impact on gametocyte prevalence or infectivity of individuals. Irrespective of whether vaccines based on asexual blood-stage antigens can reduce sexual stage parasite densities and reduce transmission, combinations of asexual blood-stage vaccines with classical TBVs will enable development of VIMT that provide direct benefit to vaccine recipients by providing protection against clinical disease in addition to reducing transmission.

### Targeting the Vector to Reduce Malaria Transmission

As described earlier, *Plasmodium* parasites have an obligatory development stage in the mosquito during which zygotes transform into ookinetes that traverse the midgut epithelium to establish oocysts on the outer wall of the midgut. Attachment and invasion of the midgut epithelium requires specific interactions between ookinete surface proteins and midgut receptors. A set of conserved “invasion receptors” on the midgut of diverse Anopheline species are used by *Plasmodium* ookinetes to attach to the midgut epithelium [31]. Antibodies directed against such receptors have been shown to block development of oocysts in membrane-feeding transmission-blocking assays [31]. A vaccine based on such conserved vector antigens should be effective against all species of *Plasmodium* and obviate the need to develop separate vaccines for different *Plasmodium* species. Moreover, since such vaccines target vector antigens, parasite strain diversity, which has been a major problem for malaria vaccine development, will be overcome. Such novel strategies will require significant fundamental research to understand vector-parasite interactions [32].

### Host-Parasite and Vector-Parasite Interactions

*Plasmodium* sporozoites invade human hepatocytes in a two-step process. In the first step, sporozoites pass through multiple hepatocytes by rupturing the plasma membrane of target hepatocytes [33]. After traversing multiple hepatocytes, sporozoites finally invade target hepatocytes by forming a parasitophorous vacuole where they multiply and differentiate into merozoites. Identification of key parasite proteins that mediate the two-step invasion process could provide functional targets for intervention. Sporozoite surface proteins such as the circumsporozoite protein (CSP) and thrombospondin-related protein (TRAP) have been shown to play a role in hepatocyte binding and invasion [34]–[37]. Both proteins contain functional cysteine-rich regions that share homology with thrombospondin and that mediate attachment to hepatocyte receptors. Antibodies targeting such functional regions can block hepatocyte invasion. Vaccines that elicit high-titer long-lasting antibodies against such functional domains might reduce the prevalence of blood-stage infection effectively. Similarly, antibodies targeting merozoite antigens such as the 175-kD erythrocyte binding antigen (EBA175) [38]–[41], Duffy binding protein [42], or PfRH proteins [43], which mediate critical interactions with erythrocyte receptors, can inhibit multiplication of blood-stage parasites. Ookinete antigens that interact with the midgut wall to mediate traversal may also be useful as recombinant malaria vaccine candidates that block parasite transmission by mosquitoes.

Because processes such as host cell invasion involve multiple steps, some of the processes highlighted above may be mediated by multiple pathways that are redundant. As a result, effective inhibition of host invasion by parasites may require targeting of a combination of receptor-ligand interactions that mediate invasion. A clear understanding of the sequence of events and functional roles of different receptor-ligand interactions will be critical for the development of vaccines that target multiple steps to provide synergistic inhibition of invasion and parasite multiplication at different stages of the parasite life cycle.

It will also be important to develop functional assays that can be used to evaluate antibody responses against the parasite antigens that mediate host cell invasion and transmission to mosquitoes. These functional assays may directly test the inhibitory activity of antibodies elicited by vaccine candidates against the biological processes themselves or may be reduced to biophysical or biochemical assays in which antibodies are tested for inhibition of functions such as receptor binding or proteolytic cleavage that are known to mediate the biological processes. Harmonization of such assays is important so that results from different research groups are comparable and to facilitate decision making for down-selection of vaccine candidates during preclinical and clinical development. Currently, there are no clear correlates of immunity against pre-erythrocytic and blood-stage parasites. Immuno-assays can be validated only once a vaccine demonstrates efficacy in a clinical trial. Once an immune correlate for protection is identified, it can be used for decision making in clinical development.

### Vaccine Delivery Systems and Adjuvants

The development of subunit vaccines will require the use of potent adjuvants and/or efficient vaccine delivery systems to elicit robust and sustainable immune responses. The unavailability of a wide range of potent adjuvants with a proven safety record in humans has been a bottleneck in the development of recombinant protein–based vaccines for malaria. Better understanding of mechanisms that activate the innate immune system might enable the design of adjuvants that elicit potent immune responses. Alternative methods to deliver antigens such as the use of virus-like particles or prime-boost strategies that use combinations of different viral vectors (e.g., recombinant adenovirus and modified vaccine virus–based vectors) or viral vectors and recombinant proteins have provided effective means to elicit potent immune responses [44], but further research on vaccine delivery systems is urgently required for development of effective malaria vaccines.

When the VIMT include multiple components, it will be important to develop formulations or delivery systems that are compatible with each component. A clear understanding of the correlates of protective immunity elicited by each component may allow the identification and development of a compatible delivery system or adjuvant formulation for the combination vaccine. Analysis of candidate vaccine–elicited immune responses in functional assays will allow optimization of compatible formulations. Importantly, development of multicomponent VIMT may require collaboration between researchers who have developed the individual components. It will be important to develop innovative licensing arrangements that ensure accessibility of each component for commercial development of such multicomponent VIMT.

### Understanding Transmission Dynamics and Population Biology of Malaria Parasites

As campaigns to reduce transmission of malaria are successful, it will be necessary to understand the changes in parasite population dynamics and population structure. In particular, it will be desirable to determine whether specific parasite strains dominate as the transmission pattern changes and whether this has implications with regard to antigenic diversity or parasite virulence. Field trials with *P. falciparum* blood-stage vaccines have provided evidence for allele-specific protection, which suggests that large-scale immunization may lead to the selection of “vaccine-resistant” parasites that can escape immune responses elicited by the vaccine [45]. A second important question is to determine whether reemergent parasites have been introduced from an outside source or whether they are parasites that have escaped control measures. These two options have very different implications for intervention strategies during the pre-elimination stage. Tools to track such parasites will be useful for surveillance as control efforts move towards eradication.

### Measuring Malaria Transmission Rates

A key to the evaluation of vaccines that block transmission will be the measurement of transmission. The anticipated clinical outcome of vaccination will be the reduction of transmission in the community. It is therefore necessary to develop robust and readily usable tools to evaluate transmission levels in various epidemiological settings ranging from high transmission areas to areas of very low prevalence and transmission. In particular, as various malaria control measures are introduced, the transmission dynamics will change and robust evaluation of transmission will be challenging. Harmonization of existing tools for measurement of transmission rates is a high priority [46],[47].

It is particularly important to be able to measure the effect on infectivity of an individual after vaccination with either a pre-erythrocytic or a blood-stage vaccine, and to understand the relation of this result to an effect on transmission in the community. Clinical efficacy trials of such vaccines have tended to focus on their impact on blood-stage infection or clinical disease; the impact of such vaccines on transmission remains to be determined. An important aspect of strategic thinking around malaria vaccines in years to come will be a greater emphasis on the evaluation of the impact of all classes of vaccines on transmission.

A second priority is the development of markers that define the infectivity of an individual for mosquitoes. These markers could include bioassays, serological parameters, or molecular markers. There is a need for robust models that predict the relationship of rates of individual infectivity to transmission at the community level in different epidemiological settings. Once this relationship is established, such markers could be used as surrogates of vaccine efficacy on transmission at the population level.

## Strategies for Product and Clinical Development of VIMT

### Product Development Based on TPP

**Figure 1:**
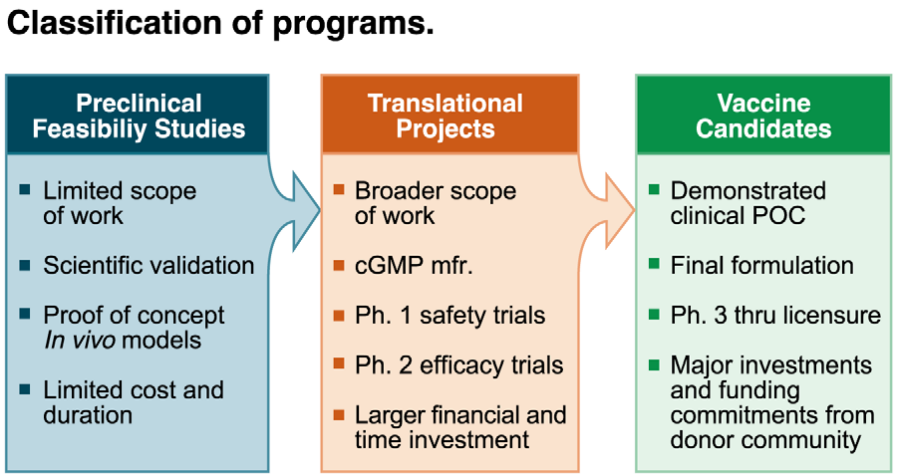
Classification of programs. Image credit: Fusión Creativa.

Once TPPs are defined, they should be used to guide product development and evaluate the project in terms of achieving desired goals set for the vaccine candidates. It is important to understand where the project stands in terms of development. Terminology should be used appropriately and be in line with the development phase of the product (Figure 1).

Preclinical feasibility studies are conducted first to validate the scientific rationale for vaccine design. At this stage of the project, questions have to be asked that address issues such as whether the project is likely to achieve the final desired TPP. Numerous preclinical feasibility studies may be undertaken to assess a variety of antigens, adjuvants, and delivery systems. Importantly, immune responses with the experimental vaccine produced at pilot scale need to be evaluated in animal models, preferably using functional assays, to validate the concept and progress it to a translational project stage.

For the translational stage, a significant investment of resources is necessary, not least because the prototype vaccine must be produced under current good manufacturing practices. Thus, only the most promising approaches can be moved into this and later stages of development. The translational project, which will have a set of precise go/no-go milestones, drives a research program of relevance to public health from the preclinical phase, through phase I trials to evaluate safety, and into phase II trials to evaluate efficacy. A successful translational project will deliver a vaccine that should be ready for phase III trials.

A product can be considered as a vaccine candidate once its manufacturability has been established and it has undergone a successful proof-of-concept phase II efficacy trial (Figure 1). For “classical” pre-erythrocytic or asexual stage vaccines, this typically requires either a phase IIa challenge trial or an efficacy trial in an endemic country. For VIMT, proof of concept may not need to be established in a malaria-endemic setting, provided that a robust read-out measurable at the level of the individual vaccinees has been shown to predict an effect on transmission at the population level. By this stage the product is fully characterized and will not change substantially. Major investments will be required, however, to complete the development program to deliver a viable vaccine for use in public health programs. Other considerations for a successful vaccine include the requirement for WHO prequalification of the vaccine for use in developing countries, an understanding in the affected communities of the ethical and practical issues associated with a long program of testing, and a significant commitment of the donor community to provide funds to support country-wide vaccine launches.

### Clinical Development and Regulatory Strategy

A vaccine that has an effect on transmission alone may not provide direct benefit to the individual. Registration pathways for such a vaccine are therefore likely to be complex, and the licensure endpoints will require careful consideration and discussion with regulatory agencies early in the development program. If the vaccine also provides individual benefit, the regulatory pathway could well be simpler.

One approach to registration for VIMT is for phase I/II programs to focus on identification of well-tolerated and immunogenic vaccine doses and schedules across a wide age range of vaccine recipients using standard safety assessments and immunologic readouts tailored for the vaccine candidate being evaluated. Randomized, controlled phase IIb proof-of-concept studies should be designed to permit the identification of a suitable vaccine efficacy endpoint at the individual level that can be validated for use in phase III trials. This endpoint must be identified and agreed in advance with regulatory agencies. The possible endpoints might include: percent reduction in parasite prevalence, especially gametocyte prevalence; percent reduction in individual infectivity as measured by percent reduction in oocyst and sporozoite counts in membrane-feeding assays; and percent reduction in infected mosquitoes fed on vaccinated volunteers that can transmit malaria to susceptible volunteers. We recognize that such efficacy endpoints at the individual level will only be surrogates for effects on malaria transmission rates at the population level. Thus, a necessary stage after conditional registration based on surrogate efficacy data will be definitive community-scale phase IV trials, which will measure reductions in effective reproduction rate (*R*_effective_) as a postmarketing commitment.

Alternatively, some experts have argued that it should be possible to design and conduct cluster-randomized trials to evaluate the efficacy of VIMT in terms of reductions in transmission rates in malaria-endemic settings. Measurement of surrogate efficacy parameters at the individual level using robust assays in such trials may allow the identification of correlates of efficacy at the population level. Such an approach would follow the more traditional route of registering a vaccine after collecting evidence for efficacy in phase IIb/III trials. Ultimately, it will be important to study the efficacy of combination of vaccines with other interventions aimed at reducing transmission.

### Decision Making in Development of VIMT

Existing methods for measurement of transmission intensity need to be harmonized and optimized to ensure that good baseline estimates are available prior to introduction of a package of interventions such as drugs and vaccines. Thus, an essential step will be a consultation process that decides on the relative utility of assays that assess the infectiousness of individuals [48], that measure transmission-blocking activity of sera [49] raised against sexual stage or mosquito antigens, and that consider trial designs to measure the impact of vaccines targeting any life cycle stage on malaria transmission [50].

Possible trial designs include community-randomized trials that use measurement of the reduction in the proportion of gametocyte carriers, the reduction in the infectiousness of humans to mosquitoes in individually randomized controlled trials, and the reduction in infection of humans as endpoints. However, the development of an assay or trial design that could provide robust, reproducible data on vaccine impact on transmission without performing large-scale community-randomized trials would be a major step forward in increasing efficiencies and timelines.

Many questions will need to be addressed to aid decision making during development of VIMT. For example, can assays such as the membrane-feeding assay be validated to meet the requirements of the International Conference of Harmonization? If so, what level of reduced infectivity as demonstrated by this assay is likely to provide community-level reduction in infection? Questions like these need to be answered so that decisions can be made about the packages of interventions required to bring the *R*_effective_ below 1 during elimination campaigns. An assessment of existing modeling work may provide information on this sort of issue [51],[52]. Other questions that will need answering include: what population coverage and level of transmission-blocking efficacy should we require from a vaccine intervention before it is transitioned into elimination campaigns and are there assays other than the membrane-feeding assay that will be useful in measurement of infectiousness of humans (for example, nucleic acid amplification-based assays for gametocytaemia)? Ways will also need to be found to optimize mosquito-feeding experiments linked to clinical vaccine trials for decision-making purposes (see also [53]).

Importantly, every step of the vaccine development, clinical evaluation, regulatory, and implementation process for VIMT needs to focus on using the TPP for vaccines and targeting transmission rather than morbidity during decision making. In addition, it will be essential to make decisions about the need to include packages of interventions when evaluating vaccines that reduce transmission (see also [52]). Decisions will also have to be made about who should receive VIMT. In endemic regions, VIMT would be delivered to infants, preferably through the routine expanded program of immunization and through periodic campaigns to the rest of the population. In regions of low malaria transmission, it may not be necessary to immunize the entire population. Instead it may be more effective to identify and immunize individuals who are responsible for the majority of the transmission in the community.

Assessment of interruption of transmission presents novel challenges and large costs, hence every effort must be made to find and adopt the most efficient mechanism for assessing efficacy. For example, could a competent regulatory authority be provided with sufficiently compelling evidence of the biological interruption of transmission activity of a vaccine (either prevention of gamtetocyte production or effects of antisera on transmission to mosquitoes) to allow registration of a vaccine with an indication for interruption of transmission at the community level, without the requirement for large-scale community randomized trial data? As mentioned earlier, phase IV studies could then follow to provide the required safety database, and measures of community effects on transmission for implementation. Industry involvement may be critical to successfully drive such a development pathway for VIMT. It will therefore be important to engage leaders of key vaccine industries as well as regulatory agencies and ethicists from affected countries in discussions early in the development pathway.

## Conclusions

Vaccines can play a key role in multisectoral efforts to eliminate and eventually eradicate malaria. Current efforts to develop malaria vaccines are primarily focused on reducing infection rates, blocking replication of the parasite in the bloodstream, and the pathologic effects of the parasite in individuals, thereby reducing malaria morbidity and mortality in vaccinated individuals. Some of these vaccines, if highly effective, may also reduce transmission. These efforts need continued support.

For elimination, it is important to view vaccines for their potential contribution to reduction of transmission, and to support additional novel approaches to vaccines that directly target sexual and mosquito stages for use in malaria control programs. In this context, we propose the broader concept of VIMT and present an actionable research and development agenda to develop such vaccines (Box 1). We also propose that novel product development and regulatory strategies that reduce the time to market should be investigated to develop, license, and implement such vaccines.

**Box 1. Summary of the Research and Development Agenda for Vaccines**

A prioritized research and development agenda to enable the development of VIMT for use as critical components in malaria elimination efforts includes:

- Development and application of novel vaccine delivery approaches and/or adjuvants to elicit long-lasting protective efficacy that makes significant impact on malaria transmission rates under diverse epidemiological settings.
- Expansion of vaccine development efforts to cover *Plasmodium* species other than *P. falciparum*, especially *P. vivax* (including hypnozoites).
- Understanding the dynamics between multiplication of asexual stage parasites, gametocytogenesis, and malaria transmission rates at the population level.
- Development of robust assays to study functional immune responses at the individual level that can predict effect on malaria transmission at the population level and allow decision making in product development.
- Development of tools to measure malaria transmission rates, thereby facilitating clinical development of vaccines that reduce malaria transmission.

## Abbreviations

TBV: transmission-blocking vaccine
TPP: target product profile
VIMT: vaccines that interrupt malaria transmission
